# Artificial intelligence in osteoporosis assessment using CT imaging: a scoping review

**DOI:** 10.3389/fmed.2026.1779483

**Published:** 2026-02-24

**Authors:** Hanwen Cheng, Yajun Zhang, Meng Meng, Simin Liu, Yang Yang, Yuyang Ran, Yuhui Kou

**Affiliations:** 1 Department of Orthopaedic Trauma, Peking University People's Hospital, Peking University, Beijing, China; 2 Emergency Department, 731 Hospital of China Aerospace Science and Industry Group, Beijing, China; 3 Department of the Second Clinical Medical, Guangdong Medical University, Dongguan, China; 4 Department of Industrial Engineering, Tsinghua University, Beijing, China; 5 Changzhi Medical College, Changzhi, Shanxi, China

**Keywords:** artificial intelligence, computed tomography, diagnosis, osteoporosis, review

## Abstract

**Purpose:**

This scoping review aimed to systematically summarize and map current research on the application of artificial intelligence (AI) in CT-based osteoporosis assessment, with a focus on methodological approaches, anatomical target regions, and reported algorithmic performance across existing studies.

**Methods:**

PubMed, EMBASE, and Web of Science databases were searched for studies published between January 1995 and December 2025. Eligible studies applied AI, machine learning, or deep learning techniques to CT images for osteoporosis classification, bone mineral density (BMD) estimation, or fracture-risk prediction. Data extraction covered study characteristics, imaging sources, analytical workflows, and validation methods.

**Results:**

A total of 51 studies were included. Most were retrospective (84.3%) and single-center (84.3%), with nearly half conducted in China. Study objectives clustered around osteoporosis diagnosis (45.1%), opportunistic screening (39.2%), and fracture-risk prediction (15.7%). Diagnostic and screening models generally achieved high performance (AUC 0.80–0.997 and 0.781–0.99, respectively), whereas fracture-risk prediction showed more modest accuracy (AUC 0.702–0.92). Across studies, technical workflows varied widely, encompassing Hounsfield Units (HU)-based quantitative analyses, radiomics-based models, end-to-end deep learning, and multimodal approaches. Such methodological diversity, combined with inconsistent validation strategies, limits direct comparison and reduces overall generalizability.

**Conclusion:**

Current evidence shows that AI-enhanced CT can achieve diagnostic and screening performance comparable to DXA and QCT, although fracture-risk prediction still requires improvement through multimodal data integration. However, methodological heterogeneity and the lack of standardized workflows limit cross-study comparability and clinical translation. Integrating AI into routine CT pipelines may reduce screening costs, enable earlier detection and intervention, and help mitigate the global burden of osteoporosis.

## 1 Introduction

Osteoporosis is a systemic condition marked by reduced bone mineral density (BMD) and deterioration of the bone’s microstructure, leading to increased brittleness and a higher likelihood of fractures. During the period of 2017 to 2018, the age-adjusted prevalence of osteoporosis among individuals aged 50 and older was recorded at 12.6% (1). The clinical and economic impact of fractures related to osteoporosis is significant. Each year in the United States, approximately 2 million new osteoporotic fractures occur, resulting in an estimated total expenditure of $17 billion. The negative consequences of these fractures include functional limitations, chronic pain, decreased quality of life, loss of autonomy, and heightened mortality risk, particularly following hip and clinical vertebral fractures (2). Consequently, the early detection of osteoporosis is essential for prompt intervention and the prevention of osteoporotic fractures and their associated complications.

The current gold standard for diagnosing osteoporosis in clinical practice is measuring BMD using dual-energy X-ray absorptiometry (DXA). DXA utilizes two different energy beams of X-rays that pass through body tissues, measuring the attenuation of X-rays to determine BMD based on the absorption characteristics of the tissues. According to the current diagnostic criteria for osteoporosis recommended by the World Health Organization, osteoporosis is defined for postmenopausal women and men aged 50 years and older as a BMD T-score of ≤ − 2.5, as measured by DXA at the lumbar spine, hip, or distal radius. This indicates a bone density that is 2.5 standard deviations or more below the mean value for young adults (3). However, DXA measurements reflect a two-dimensional assessment of bone mineral content and fail to account for geometric differences and structural variations, making them less sensitive to changes in bone strength. Furthermore, clinical practice has shown that DXA results can be affected by factors such as bone spurs, internal fixation devices, and ectopic calcification, leading to inaccuracies in bone density readings (4). Consequently, the capability of DXA for early diagnosis and precise assessment of osteoporosis is limited.

Quantitative computed tomography (QCT) is a multi-planar, three-dimensional imaging technique for measuring bone mineral density, which allows for the macroscopic assessment of the material and structural properties of bone. It provides clinicians with more accurate descriptions of bone shape, size, and quality compared to DXA. Unlike the two-dimensional measurements offered by DXA, QCT can separately evaluate cortical and trabecular bone, offering a more comprehensive assessment of three-dimensional skeletal parameters and enabling more precise diagnosis of osteoporosis (5). This approach is particularly useful for evaluating trabecular volumetric BMD, with osteoporosis being diagnosed at levels below 80 mg/cm^3^. Low bone mass is defined by measurements ranging from 80 to 120 mg/cm^3^, following the diagnostic standards established by the American College of Radiology (ACR). However, the lack of standardized large databases that are age, sex, and ethnicity-matched limits QCT’s ability to provide personalized age-based bone assessments, restricting its application in clinical practice. This limitation is particularly significant in cases requiring comprehensive considerations of individual patient factors for osteoporosis diagnosis and risk evaluation. Furthermore, the need for specialized examinations can result in additional costs, cumbersome computational processes, and increased radiation exposure, further complicating the clinical implementation of QCT in routine practice (6).

An emerging and convenient strategy for assessing BMD involves the widespread use of conventional computed tomography (CT) scans, which do not require patients to incur additional costs for specialized bone density examinations or face increased radiation exposure. However, the complexity of image segmentation and the extraction of regions of interest hinder broad implementation (7). With the continuous integration and advancement of artificial intelligence (AI) in medical imaging, these cumbersome tasks can be streamlined. Opportunistic BMD screening utilizing CT images collected for different indications is now feasible with AI support. Integrating AI with traditional CT imaging significantly enhances the scope of osteoporosis screening and improves diagnostic accuracy. Furthermore, with increasing computational power, factors such as gender, ethnicity, and age can be incorporated into the analysis. This combination of AI and CT imaging offers a more nuanced assessment of bone health, paving the way for transformative strategies in the future diagnosis and treatment of osteoporosis.

This review aims to explore the application of AI in conjunction with CT for the diagnosis of osteoporosis. It will provide a comprehensive overview of various AI algorithms, highlighting their strengths and limitations when integrated with different types of CT imaging data. Additionally, the review will discuss potential future directions for this innovative approach, emphasizing how AI can enhance diagnostic accuracy, streamline processes, and ultimately improve patient outcomes in the field of osteoporosis management. By addressing these aspects, this review seeks to contribute valuable insights into the evolving landscape of osteoporosis diagnostics, emphasizing the role of AI as a transformative tool in clinical practice.

## 2 Materials and methods

Our protocol was developed in accordance with the Preferred Reporting Items for Systematic Reviews and Meta-Analyses Protocols (PRISMA-P) guidelines and was critically reviewed and approved by all authors (8).

To ensure that this scoping review remains comprehensive and focused, the inclusion criteria were designed to specifically capture studies applying AI techniques to CT-based osteoporosis assessment. Eligible articles were those that employed artificial intelligence, machine learning, or deep learning methods for the classification of osteoporosis or osteopenia, or for the determination of bone mineral density, using CT images of the vertebrae or other skeletal regions. Studies utilizing either conventional or low-dose CT, with or without contrast enhancement, were included.

Papers were eligible if they were published in English, involved human participants, and provided explicit information on AI-assisted CT analysis for osteoporosis assessment. Studies employing quantitative, qualitative, or mixed research designs were all considered to encompass a wide range of methodological perspectives. Articles not meeting these criteria were excluded. This selection strategy ensured that the review remained both comprehensive and targeted, facilitating a deeper understanding of how AI-driven diagnostic workflows using CT imaging can enhance the detection, classification, and management of osteoporosis in clinical and opportunistic screening contexts.

To identify all relevant studies, a comprehensive search was conducted across PubMed, EMBASE, and Web of Science databases covering the period from January 1995 to October 2025. The initial search strategy was developed by the review team and refined through iterative discussions to ensure both sensitivity and specificity.

The following search string was used for PubMed: (“CT”[tiab] OR “computed tomography”[tiab]) AND (“artificial intelligence”[tiab] OR “AI”[tiab] OR “machine learning”[tiab] OR “deep learning”[tiab]) AND (“osteoporosis”[tiab] OR “bone mineral density”[tiab] OR “BMD”[tiab]).

Equivalent search strategies, adapted to the syntax of each database, were applied to EMBASE and Web of Science.

**Figure 1:**
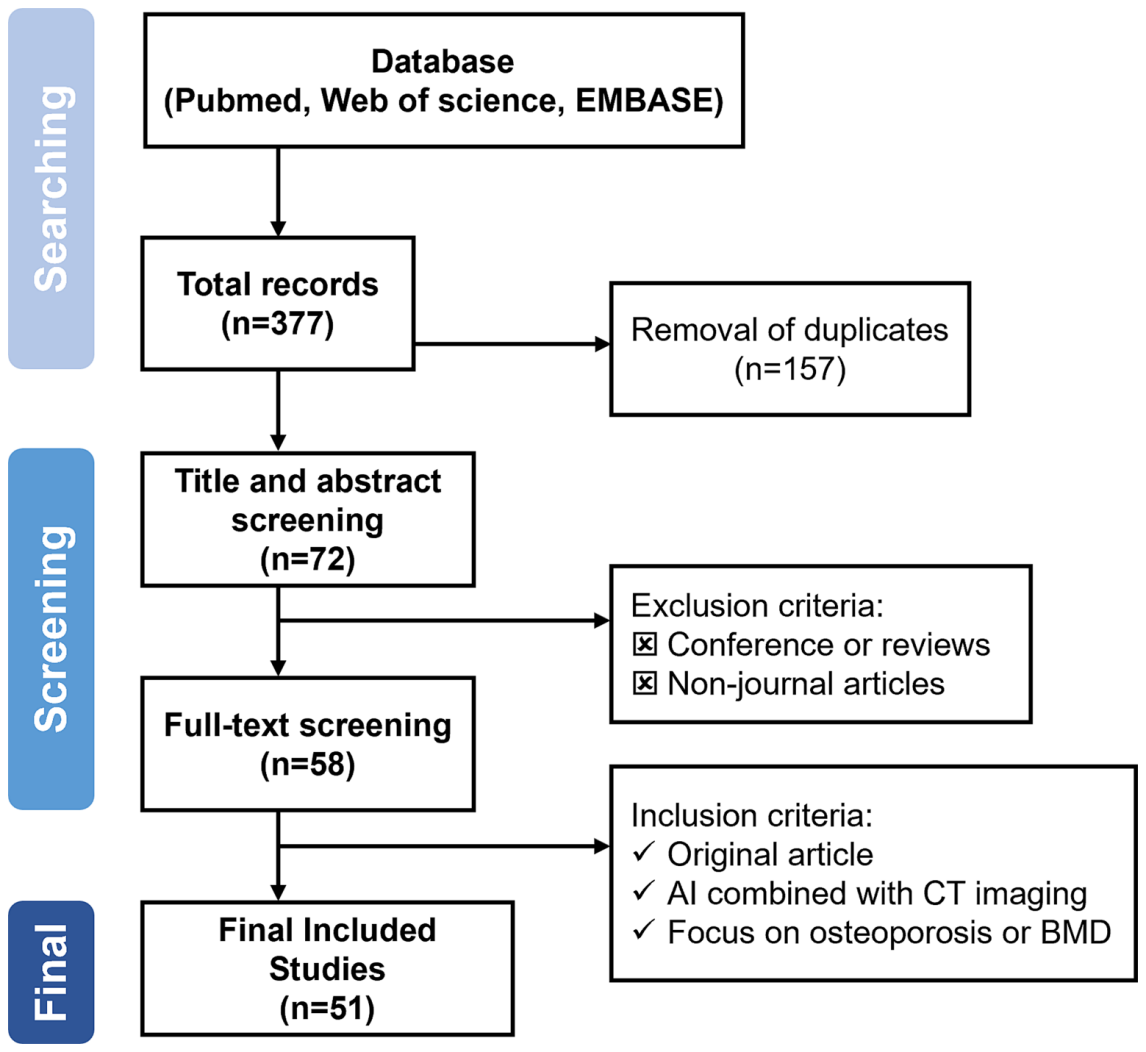
Literature search flow and final inclusion criteria for this review.

Initially, all search results were imported into EndNote X9 software, where duplicate articles were identified and removed. Subsequently, reviewers assessed the titles and abstracts to further filter the studies. The reviewers then read the full texts to determine the final studies to be included in the review. During this process, any relevant articles that emerged were also considered for inclusion. In cases where disagreements arose regarding the study type, data type, or other extracted information, they were resolved through discussion. The literature screening process for this study is illustrated in Figure 1.

**Figure 2:**
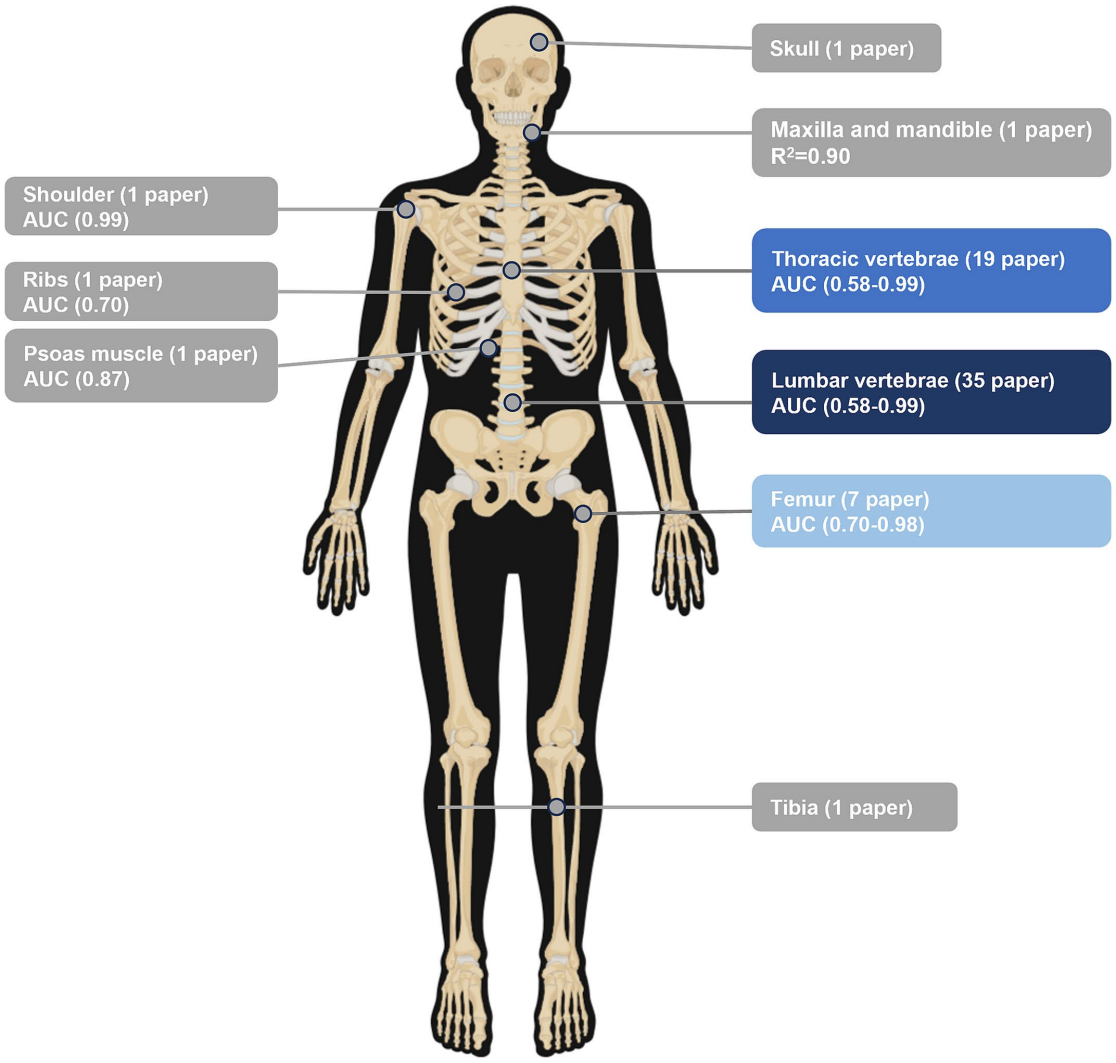
The distribution of the regions of interest selected for all the included CT images in the study.

The primary objective of this review is to explore the application of AI combined with CT imaging in the diagnosis of osteoporosis. Accordingly, we designed tables and figures to systematically compare and discuss the standardized workflows, application contexts, and performance of all included studies. This approach provides a comprehensive overview of the current landscape and effectiveness of AI in osteoporosis diagnosis (see Figure 2).

## 3 Results

### 3.1 Search results

Per the PRISMA guidelines, our review process began with the identification of 377 records through database searches (Figure 1). After removing 157 duplicate records, the remaining articles underwent title and abstract screening. During this stage, several studies were excluded because they were reviews, conference papers, or other non-journal publications. A total of 72 articles were retrieved for full-text assessment. Following a detailed evaluation, 51 studies were found to meet the inclusion criteria, specifically focusing on the application of AI techniques to CT imaging for osteoporosis or bone mineral density analysis. These eligible studies were included in the final review and subjected to comprehensive data extraction and thematic synthesis.

### 3.2 Study characteristics overview

The scoping review ultimately included 51 studies, which are systematically summarized in Table 1, detailing their objectives, research design, imaging sources, and participant demographics. Studies were published between 2019 and 2025 and originated from diverse geographic regions, with Asia—particularly China—accounting for nearly half of all publications (49%). The majority of investigations were single-center and retrospective in nature (84.3%), whereas only a limited proportion incorporated multicenter cohorts or external validation (15.7%), highlighting a general lack of large-scale validation across institutions.

**Table 1:** Overview of study characteristics and extracted information.

Authors and year		Main objectives	Research centers	Country	CT image sources	Participants (male/female)	Age range (years)
Wu et al. (9)	2025	OP diagnosis	Single-center	China	Chest CT	553 (353/200)	M 50 ± 11
							F 49 ± 10
Li et al. (10)	2025	OP diagnosis	Single-center	China	Lumbar spine CT	120 (48/72)	20–79
Wu et al. (11)	2025	OP screening	Single-center	China	Chest CT	7,713 (3,544/4169)	47–61
Adarve et al. (12)	2025	OP diagnosis	Multicenter	Spain	Abdominal CT	58 (11/47)	49–65
Paukovitsch et al. (13)	2025	OP diagnosis	Multicenter	Germany	Chest and abdominal CT	207 (124/83)	71–84
Liu et al. (14)	2025	OP diagnosis	Single-center	China	Abdominal CT	509 (272/237)	58 ± 12
Du et al. (15)	2025	OP diagnosis	Single-center	China	Low-dose abdominal CT	456 (217/239)	50–91
Petraikin et al. (16)	2025	OP diagnosis	Multicenter	Russia	Chest CT	1888 (733/1155)	66 ± 8
Imani et al. (17)	2025	OP diagnosis	Multicenter	Australia, USA, China	Hip CT	300 (300/0)	73 ± 6
Zhou et al. (18)	2025	OP diagnosis	Single-center	China	Chest CT	551 (397/154)	22–81
Yuan et al. (19)	2025	Fracture risk prediction	Single-center	China	Hip CT	254 (NA)	M 83 ± 8
							F 80 ± 9
Li et al. (10)	2025	OP diagnosis	Single-center	China	Chest CT	987 (396/591)	30–89
Guenoun et al. (20)	2025	Fracture risk prediction	Single-center	France	Hip CT	100 (28/72)	76 ± 10
Fang et al. (21)	2024	OP screening	Single-center	China	Chest CT	488 (188/300)	Average age > 62
Cheng et al. (22)	2023	OP diagnosis	Single-center	China	Lumbar spine CT	154 (51/103)	Average age > 58
Liu et al. (23)	2022	OP screening	Single-center	China	Clinical examination	1,508 (705/803)	Average age > 55
Huber et al. (24)	2024	OP screening	Single-center	USA	Chest CT	3,708 (1990/1718)	64 ± 7
Dzierzak et al. (25)	2022	OP diagnosis	Single-center	Poland	Lumbar spine CT	100 (41/59)	53–77
Huang et al. (26)	2022	OP screening	Single-center	China	Clinical examination	172 (64/118)	Average age > 62
Mu et al. (27)	2021	Bone quality assessment	Single-center	China	Lumbar spine CT	56 (35/21)	45–69
Wu et al. (11)	2024	OP screening	Single-center	China	Low-dose chest CT	3,312 (1772/1540)	All age
Rühling et al. (28)	2022	OP screening	Single-center	Germany	Abdominal CT	193 (145/48)	62 ± 14
Pan et al. (29)	2023	OP diagnosis	Single-center	China	Chest CT	1,175 (773/402)	Not mention
Pan et al. (30)	2020	OP screening	Single-center	China	Chest CT	374 (196/178)	50–88
Sebro et al. (31)	2024	OP screening	Single-center	USA	Shoulder CT	194 (24/170)	69 ± 9
Breit et al. (32)	2023	OP screening	Single-center	Switzerland	Chest CT	109 (19/90)	67 ± 12
Tang et al. (33)	2021	OP screening	Single-center	China	Spine CT	213 (38/175)	17–84
Guha et al. (34)	2024	OP diagnosis	Single-center	USA	Different CT	20 (10/10)	26.2 ± 4.5
Li et al. (35)	2022	OP diagnosis	Single-center	China	Different CT	716 (416/300)	50–97
Uemura et al. (36)	2023	OP screening	Multicenter	Japan	Hip CT	857 (189/668)	70 ± 16.8
Uemura et al. (37)	2023	OP diagnosis	Single-center	Japan	Spine CT	59 (18/41)	66 ± 17
Uemura et al. (38)	2022	OP diagnosis	Single-center	Japan	Hip CT	123 (0/123)	62 ± 10
Tong et al. (39)	2024	OP screening	Single-center	China	Chest CT	1,508 (803/705)	M 63 ± 12
							F 60 ± 11
Oh et al. (40)	2024	OP screening	Single-center	South Korea	Abdominal CT	268 (122/146)	58 ± 12
Oh et al. (41)	2024	OP screening	Single-center	South Korea	Different CT	112 (17/68)	61 ± 12
Yoshida et al. (42)	2023	OP diagnosis	Single-center	Japan	Lumbar spine CT	402 (91/311)	49–74
Summers et al. (43)	2011	OP diagnosis	Single-center	USA	Lumbar spine CT	475 (0/475)	42–79
Liu et al. (44)	2024	Fracture risk prediction	Single-center	USA	Hip CT	6,926 (2,648/4278)	70 ± 12
Nam et al. (45)	2019	OP diagnosis	Single-center	South Korea	Spine CT	70 (50/20)	21–95
Naghavi et al. (46)	2023	OP diagnosis	Single-center	USA	Chest CT	165 (86/79)	69 ± 9
Fang et al. (47)	2021	OP screening	Single-center	China	Different CT	1,449 (644/805)	51 ± 14
Yang et al. (48)	2022	OP screening	Single-center	China	Chest CT	1,046 (679/367)	Not mention
Yasaka et al. (49)	2020	OP diagnosis	Single-center	Japan	Lumbar spine CT	183 (112/83)	60 ± 15
Lim et al. (50)	2021	OP screening	Single-center	South Korea	Abdomen-pelvic CT	500 (70/430)	50–96
Zhang et al. (51)	2019	Fracture risk prediction	Single-center	China	Lumbar spine CT	80 (unknown)	Not mention
Yong et al. (52)	2021	OP diagnosis	Single-center	South Korea	Cranial CT	NA	Not mention
Bodden et al. (53)	2024	OP diagnosis, Fracture risk prediction	Single-center	Germany	Chest-abdominal CT	121 (77/44)	65 ± 8
Tang et al. (54)	2023	Fracture risk prediction	Single-center	China	Chest CT	2076 (1,309/767)	47–62
Savage et al. (55)	2020	OP diagnosis	Single-center	USA	Chest CT	65 (8/57)	67 ± 10
Park et al. (56)	2023	OP diagnosis	Single-center	South Korea	Cone-Beam CT	30 (18/12)	21–80
Naghavi et al. (57)	2023	OP diagnosis	Multicenter	USA	Cardiac CT	5,785 (2,748/3037)	62 ± 10

NA, not applicable. OP, osteoporosis.

Study objectives clustered around three main topics: osteoporosis diagnosis (45.1%), opportunistic screening (39.2%) and fracture-risk prediction (15.7%). Chest CT (33.3%), lumbar spine CT (15.7%) and abdominal CT (11.8%) were the most frequently utilized imaging sources, reflecting the increasing interest in opportunistic use of routinely acquired CT data. Participant cohorts were largely adult and elderly populations (typical age ranges spanning roughly from the third to the ninth decades), with a predominance of female participants, consistent with the epidemiology of osteoporosis.

#### 3.2.1 Osteoporosis diagnosis: high accuracy accompanied by pronounced technical heterogeneity

AI-enhanced CT demonstrated consistently high accuracy for osteoporosis diagnosis, with reported AUC values ranging from 0.80 to 0.997. Overall, 78.1% of studies achieved an AUC ≥ 0.9 (e.g., (10); (13)). Diagnostic performance was highest when lumbar spine ROIs were used, particularly T12–L3 or L1–L4 segments, where AUC values ranged from 0.903 to 0.997. No substantial performance difference was observed between HU-based quantitative workflows and radiomics-based approaches, with HU-based methods showing strong quantitative agreement (*R*^2^ > 0.98) and radiomics-based models achieving comparable classification accuracy (AUC ≈ 0.96). In addition to spinal ROIs, the proximal femur also served as an effective alternative diagnostic site; for example, Du et al. (15) reported an AUC ≥ 0.9, supporting the feasibility of multi-site CT-based osteoporosis diagnosis.

However, several sources of technical and population-related heterogeneity were evident. Diagnostic accuracy was strongly influenced by ROI extent, as studies using narrowly defined ROIs such as L1–L2 (18) reported substantially lower performance (AUC = 0.80) compared with those employing broader ROIs (e.g., T12–L4). Heterogeneity in reference standards further limited cross-study comparability: while 68.6% of studies used DXA and 21.6% used QCT, even identical ROIs (L1–L3) yielded different performance when validated against DXA [AUC = 0.925; (45)] versus QCT [AUC = 0.874; (16)]. In addition, marked population bias was observed, with 82.4% of studies focusing on individuals aged ≥50 years and only 9% including younger populations, often with small sample sizes [e.g., *n* = 20 (34)], leaving diagnostic applicability in younger or healthy populations insufficiently characterized.

#### 3.2.2 Opportunistic screening: efficiency gains accompanied by context-dependent limitations

Opportunistic screening based on chest or abdominal CT accounted for 39.2% of the included studies. Its core advantage is the absence of additional radiation exposure. Fully automated HU-based models [e.g., (11, 24)] enabled large-scale screening (*n* > 3,000), achieving AUC values ranging from 0.781 to 0.99. Screening based on non-traditional sites, such as shoulder CT (31), also demonstrated high accuracy (AUC = 0.988), extending opportunistic screening beyond the axial skeleton.

However, screening performance was strongly dependent on imaging coverage. Chest CT typically includes only T7–L1 vertebrae, whereas abdominal CT primarily captures L1–L4, which limits direct model transferability across different screening scenarios. In addition, the impact of low-dose CT on screening accuracy remains insufficiently characterized. Only 5 of 51 studies employed low-dose CT protocols; although Du et al. (15) reported an AUC of 0.96, no direct comparison with standard-dose CT was conducted to evaluate feature stability. Finally, a trade-off between automation efficiency and interpretability was evident. Approximately 75% of screening studies adopted end-to-end CNN-based models [e.g., (28)], achieving high throughput but offering limited explanation of the imaging features underlying osteoporosis detection, which may constrain clinical acceptance.

#### 3.2.3 Fracture risk prediction: multimodal advantages and performance bottlenecks

Fracture risk prediction represented the smallest proportion of included studies (15.7%) and demonstrated relatively modest performance, with reported AUC values ranging from 0.702 to 0.92. A majority of these studies (62.5%) adopted multimodal models integrating CT-derived imaging features with clinical variables. Models using site-matched ROIs showed comparatively better performance; for example, Yuan et al. (19) used proximal femur CT to predict hip fracture risk and achieved an AUC of 0.851, supporting the value of anatomical correspondence between ROI selection and fracture outcome.

However, several limitations constrained predictive performance and robustness. Overall performance was substantially lower than that reported for osteoporosis diagnosis or screening, with only 3 of 4 studies achieving an AUC ≥ 0.8, and Liu et al. (44) reporting an AUC of 0.702 using combined femoral and lumbar ROIs. This likely reflects the multifactorial nature of fracture risk, which encompasses bone quality, fall risk, and comorbidities that were not comprehensively captured in most models. In addition, limited sample size undermined result stability: among the eight studies, only two [e.g., (20)] included more than 500 participants, while the remainder relied on small cohorts (*n* < 300) without large-scale validation. Finally, the absence of standardized fracture risk endpoints further limited comparability, as some studies predicted any fracture whereas others focused on major osteoporotic fractures, precluding direct cross-study comparison.

### 3.3 Overview of study workflows

#### 3.3.1 HU-based quantitative workflow

**Figure 3:**
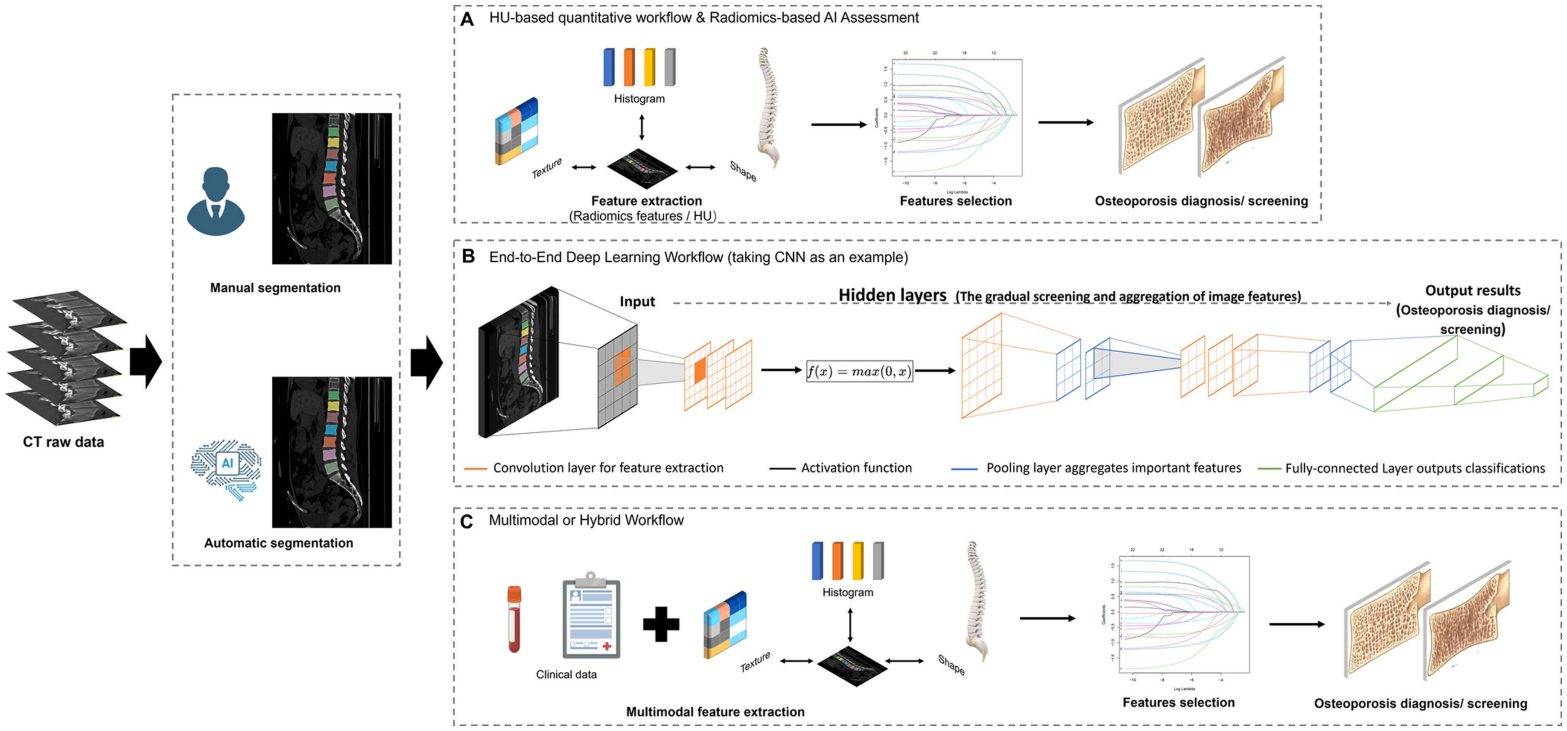
The main intelligent image-recognition methods. **(A)** Traditional machine learning based on radiomics/Hounsfield unit (HU) value, characterized by the pre-definition of imaging features. **(B)** End-to-end deep learning workflow. **(C)** A multimodal or hybrid workflow that integrates clinical features with CT-derived imaging features for comprehensive osteoporosis assessment.

This approach was used in 47.1% of the included studies (Table 2; Figure 3A). After ROI segmentation using algorithms such as U-Net or VB-Net, quantitative features—including mean and distributional Hounsfield Unit (HU) values—were directly extracted from CT images for BMD estimation or osteoporosis classification. Its core advantages include a high degree of automation and low computational cost, without the need for complex feature engineering, making it well suited for opportunistic screening using chest or abdominal CT [e.g., Wu et al. (9) achieved BMD prediction with an *R*^2^ > 0.98 using T12–L2 ROIs on chest CT]. However, this approach primarily captures overt bone density loss and is relatively insensitive to early microstructural bone alterations.

**Table 2:** Technical details of the included studies.

Authors and year		Selected area	Selection of ROI		CT features utilization	Reference standard	Performance
Wu et al. (9)	2025	T7–L1	U-Net	Automated	HU	DXA	*R*^2^ = 0.58–0.61
Li et al. (10)	2025	T12–L3	U-Net	Automated	HAP-water	QCT	AUC = 0.979
Wu et al. (11)	2025	T12–L2	3D U-Net	Automated	HU	QCT	*R*^2^>0.98
Adarve et al. (12)	2025	L1, L2	NA	Automated	Radiomics	DXA	AUC = 0.916
Paukovitsch et al. (13)	2025	T1 – L3	CNN	Automated	HU	QCT	AUC = 0.96
Liu et al. (14)	2025	L1-L3	NA	Manual	Radiomics	QCT	AUC = 0.839
Du et al. (15)	2025	Proximal femur	VB-Net	Automated	Radiomics	QCT	AUC = 0.96
Petraikin et al. (16)	2025	T11–L3	NA	Manual	HU	QCT	AUC = 0.874
Imani et al. (17)	2025	Proximal femur	NA	Manual	HU	DXA	NA
Zhou et al. (18)	2025	L1–L2	U-Net	Automated	HU	QCT	AUC = 0.8
Yuan et al. (19)	2025	Proximal femur	U-Net	Automated	Radiomics	Fracture	AUC = 0.851
Li et al. (10)	2025	T12–L2	VB-Net	Automated	HU	QCT	AUC = 0.997
Guenoun et al. (20)	2025	T1–L5	U-Net	Automated	HU	Fracture	AUC = 0.92
Fang et al. (21)	2024	T12–L4	NA	Manual	Radiomics, HU	DXA	AUC = 0.99
Cheng et al. (22)	2023	L1–L4	NA	Manual	Radiomics	DXA	AUC = 0.959
Liu et al. (23)	2022	T10–L5	VB-Net	Automated	Radiomics, HU	DXA	AUC = 0.962
Huber et al. (24)	2024	T12	Anduin	Automated	HU	DXA	AUC = 0.91
Dzierzak et al. (25)	2022	L1	U-Net	Automated	Radiomics	DXA	AUC = 0.96
Huang et al. (26)	2022	Psoas muscle	NA	Manual	Radiomics	DXA	AUC = 0.87
Mu et al. (27)	2021	L1–L4	AI-Rad	Manual	Radiomics, HU	DXA	AUC = 0.923
Wu et al. (11)	2024	T12–L2	U-Net	Automated	Radiomics	QCT	AUC = 0.98
Rühling et al. (28)	2022	T8–L2	U-Net	Automated	Radiomics	NA	AUC = 0.99
Pan et al. (29)	2023	L1	U-Net	Automated	Radiomics	QCT	AUC = 0.903
Pan et al. (30)	2020	T1–T2	U-Net	Automated	HU	QCT	AUC = 0.942
Sebro et al. (31)	2024	Humerus, Glenoid, Coracoid, Acromion, Clavicle, Ribs	NA	Manual	Radiomics, HU	DXA	AUC = 0.988
Breit et al. (32)	2023	Thoracic	3D CNN	Automated	Radiomics	DXA	AUC = 0.93
Tang et al. (33)	2021	L1	U-Net	Automated	Radiomics	DXA	AUC = 0.917
Guha et al. (34)	2024	Distal Tibia	3D GAN-CIRCLE Model	Semi-automated	Radiomics	DXA	NA
Li et al. (35)	2022	L1–L3	NA	Manual	Radiomics	DXA	NA
Uemura et al. (36)	2023	Proximal femur and L1–L4	U-Net	Automated	Radiomics	DXA	AUC = 0.976
Uemura et al. (37)	2023	L1–L4	U-Net	Automated	Radiomics	DXA	AUC = 0.941
Uemura et al. (38)	2022	Proximal femur	U-Net	Automated	Radiomics	DXA	AUC = 0.97
Tong et al. (39)	2024	T10–L5	VB-Net	Automated	Radiomics, HU	DXA	AUC = 0.97
Oh et al.(40)	2024	L1–L4	U-Net	Semi-Automated	Radiomics, HU	DXA	AUC = 0.781
Oh et al. (41)	2024	L1–L2	U-Net	Automated	Radiomics, HU	DXA	AUC = 0.847
Yoshida et al. (42)	2023	L1–L4	U-Net	Automated	Radiomics	DXA	AUC = 0.921
Summers et al. (43)	2011	L1–L5	Rad Companion	Automated	Radiomics, HU	DXA	*R*^2^ = 0.98
Liu et al. (44)	2024	Femur, lumbar vertebra, etc.	U-Net	Automated	Radiomics, HU	DXA	AUC = 0.702
Nam et al. (45)	2019	L1–L3	U-Net	Automated	HU	DXA	AUC = 0.925
Naghavi et al. (46)	2023	Thoracic and lumbar	U-Net	Automated	Radiomics	DXA	*R*^2^ = 0.95
Fang et al. (47)	2021	L1–L4	U-Net	Automated	Radiomics	QCT	*R*^2^ = 0.96
Yang et al. (48)	2022	Thoracic and lumbar	AI-Rad Companion	Automated	Radiomics, HU	DXA	AUC = 0.831
Yasaka et al. (49)	2020	L1 – L5	CNN	Automated	Radiomics	DXA	AUC = 0.970
Lim et al. (50)	2021	Left proximal femur	NA	Manual	Radiomics	DXA	AUC = 0.96
Zhang et al. (51)	2019	L1	NA	Manual	Radiomics	NA	*R*^2^ = 0.97
Yong et al. (52)	2021	Human Skull	QCBCT-NET	Automated	Radiomics, HU	QCT	NA
Bodden et al. (53)	2024	L1–L4	CNN	Automated	Radiomics	DXA	NA
Tang et al. (54)	2023	Ribs	U-Net	Automated	Radiomics	DXA	AUC = 0.706
Savage et al. (55)	2020	Thoracic vertebra	AdaBoost	Automated	HU	DXA	NA
Park et al. (56)	2023	Maxilla, Mandible	QCBCT-NET	Automated	Radiomics	QCT	*R*^2^ = 0.9
Naghavi et al. (57)	2023	Thoracic vertebra	U-Net	Automated	Radiomics	NA	*R*^2^ = 0.85

NA, not applicable; CNN, convolutional neural networks; DXA, dual-energy X-ray absorptiometry; QCT, quantitative computed tomography; HU, Hounsfield unit; HAP, hydroxyapatite; T, thoracic spine; L, lumbar spine.

#### 3.3.2 Radiomics-based workflow

This approach was adopted in 70.6% of the included studies. Following ROI segmentation, multidimensional radiomic features—including texture, morphological, and intensity-based statistics—were extracted and modeled using conventional machine learning algorithms such as support vector machines and random forests (Figure 3A). Its main advantage lies in capturing microstructural bone alterations, including trabecular heterogeneity and connectivity; for example, Cheng et al. (22) achieved early osteopenia identification using L1–L4 lumbar ROIs with an AUC of 0.959. However, the workflow involves higher procedural complexity and is less suitable for large-scale screening applications.

#### 3.3.3 End-to-end deep learning workflow

These studies employ CNNs, 3D U-Nets, or Transformer-based models to directly learn predictive features from raw CT images for BMD estimation or osteoporosis risk stratification, without explicit feature extraction (Figure 3B). This end-to-end approach offers higher automation, reduced human intervention, and improved cross-device generalizability, but the interpretability of features associated with BMD changes or fracture risk is limited.

#### 3.3.4 Multimodal or hybrid workflow

This workflow was primarily applied in fracture risk prediction studies, where CT-derived imaging features (HU values and/or radiomic features) were integrated with clinical variables such as age, sex, BMI, and fracture history to construct predictive models (Figure 3C). This approach captures the combined risk arising from bone quality and overall health status; for example, Guenoun et al. (20) achieved an AUC of 0.92 for vertebral fracture prediction by integrating thoracic CT HU values with clinical history. However, this workflow is the most complex and exhibits greater heterogeneity than single-modality models.

### 3.4 Drivers of study-level heterogeneity and performance variability

**Figure 4:**
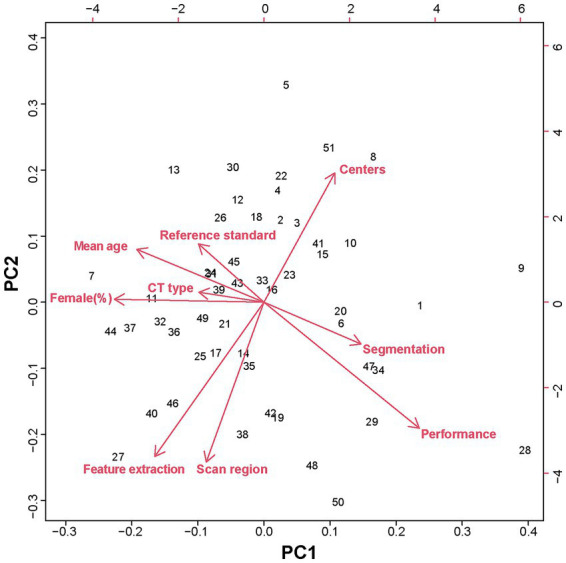
Principal component analysis (PCA) of study-level heterogeneity. PCA based on nine key study-level variables across the included studies. The first two principal components (PC1 and PC2) are shown. Red arrows represent the loading vectors of individual variables, indicating the direction and magnitude of their contributions to variance in the PCA space, including number of centers, mean age, proportion of female participants, CT modality, anatomical scan region, segmentation strategy, feature extraction method, reference standard, and model performance. The relative orientation and length of the arrows reflect correlations among variables and their influence on study heterogeneity.

To identify the sources of heterogeneity across studies, principal component analysis (PCA) was performed on nine key variables from the 51 included studies: number of centers, mean age, proportion of female participants, CT modality, anatomical scan region, segmentation strategy, feature extraction method, reference standard, and model performance (Figure 4). The first two principal components jointly explained 35.55% of the total variance (PC1: 18.78%; PC2: 16.77%).

PC1 was primarily driven by model performance (loading = 0.49), proportion of female participants (loading = −0.47), and mean age (loading = −0.40), indicating that studies involving younger populations with a lower female proportion tended to report higher model performance.

PC2 was mainly influenced by anatomical scan region (loading = −0.53), feature extraction strategy (loading = −0.51), and number of centers (loading = 0.43). These findings suggest that single-center studies focusing on core skeletal sites such as the lumbar spine or hip and employing combined HU and radiomics features achieved higher performance, whereas multicenter studies—often designed to reflect real-world clinical scenarios such as opportunistic screening using chest CT—tended to analyze non-core regions and simpler feature sets, resulting in slightly lower performance but improved generalizability.

Together, these findings quantitatively demonstrate that population characteristics, anatomical focus, and methodological choices jointly shape reported model performance across studies.

## 4 Discussion

### 4.1 Core findings and clinical implications

This scoping review synthesizes 51 studies and demonstrates that AI-enhanced CT imaging has progressed from proof-of-concept toward task-specific clinical utility in osteoporosis assessment. Rather than algorithmic complexity alone, model performance across studies was primarily determined by the alignment between clinical task (diagnosis, screening, or risk prediction), anatomical target, and analytical workflow, a pattern consistently supported by both subgroup analyses and PCA findings.

Osteoporosis diagnosis represented the most technically mature application. Across studies, diagnostic performance was consistently high (AUC 0.80–0.997), particularly when lumbar spine ROIs spanning L1–L4 or T12–L3 were used. This anatomical advantage is biologically plausible, as the lumbar spine contains a high proportion of trabecular bone, which is metabolically active and sensitive to early osteoporotic changes (58). Importantly, both HU-based quantitative workflows and radiomics-based approaches achieved comparable diagnostic accuracy, but through fundamentally different mechanisms. HU-based models predominantly captured global bone mineral density loss and showed excellent agreement with DXA and QCT (*R*^2^ > 0.98), making them well suited for standardized diagnosis and large-scale deployment. In contrast, radiomics-based models leveraged texture and heterogeneity features to characterize trabecular microarchitecture, enabling improved sensitivity for early osteopenia where mean HU values alone may remain within normal ranges (59). These findings suggest that HU- and radiomics-based workflows should be viewed as complementary rather than competing strategies, optimized for different stages of skeletal deterioration.

Opportunistic screening emerged as a distinct translational pathway rather than a simple extension of diagnostic modeling. Nearly 40% of included studies repurposed chest or abdominal CT scans acquired for unrelated clinical indications, confirming the feasibility of embedding osteoporosis assessment into routine imaging workflows without additional radiation exposure. However, screening scenarios imposed stricter constraints on model design, including incomplete vertebral coverage, heterogeneous scan protocols, and the need for full automation. Accordingly, HU-based and end-to-end deep learning models predominated, prioritizing robustness and scalability over maximal accuracy. The observation that non-traditional sites, such as shoulder CT, could also yield high screening performance further challenges the conventional reliance on axial skeletal regions and underscores the adaptability of CT-based AI when anatomical context is carefully considered. Nevertheless, performance variability across scan regions highlights that opportunistic screening models are inherently context-dependent and cannot be indiscriminately transferred across imaging protocols.

In contrast to diagnosis and screening, fracture risk prediction remained the least mature and most heterogeneous application. Although multimodal models integrating imaging features with clinical variables consistently outperformed imaging-only approaches, overall performance was modest (AUC 0.70–0.92). This gap reflects a fundamental conceptual distinction: fracture risk is a prospective, multifactorial outcome driven not only by bone density and microstructure, but also by fall mechanics, muscle strength, neuromuscular coordination, and comorbidities (60, 61). CT-derived skeletal features capture only one component of this risk cascade, explaining why even anatomically matched, high-quality imaging fails to achieve diagnostic-level accuracy. These findings suggest that fracture prediction should be framed not as an extension of osteoporosis diagnosis, but as a distinct modeling problem requiring broader phenotypic integration.

### 4.2 Evidence-based drivers of heterogeneity across studies

Beyond task-specific differences, substantial heterogeneity was observed across studies, and the PCA analysis provides quantitative insight into its underlying drivers. The first principal component linked model performance with population characteristics, particularly age distribution and sex composition. Studies involving younger cohorts and lower proportions of female participants consistently reported higher accuracy, a pattern likely attributable to reduced imaging confounders. In elderly populations—especially postmenopausal women—degenerative changes, osteophytes, vertebral deformities, and prior fractures introduce complex imaging patterns that challenge both density-based and texture-based models (62). This observation highlights that reported performance metrics cannot be interpreted independently of population context.

The second principal component reflected methodological trade-offs between anatomical focus, feature strategy, and study scale. Single-center studies targeting core skeletal regions (lumbar spine or proximal femur) and employing richer feature representations tended to achieve higher peak performance. In contrast, multicenter studies—often designed to approximate real-world opportunistic screening—frequently analyzed non-core regions and relied on simpler features, resulting in slightly lower accuracy but improved generalizability. These findings reveal an inherent tension between methodological optimization and clinical realism, suggesting that maximal performance achieved under controlled conditions may not translate directly to routine practice.

Geographical and study-design bias further contributed to heterogeneity. Nearly half of the included studies were conducted in China, and most adopted single-center retrospective designs, reflecting the availability of large-scale CT archives and centralized healthcare systems. While this setting facilitates rapid model development and high internal performance, it may underrepresent interethnic variability and real-world practice patterns, potentially inflating reported accuracy. This imbalance underscores the need for prospective, multicenter, and cross-ethnic validation to ensure robust generalizability.

Additional heterogeneity arose from inconsistent ROI definitions, segmentation strategies, and reference standards. Even when identical vertebral levels were analyzed, validation against DXA versus QCT produced divergent performance estimates, reflecting fundamental differences in what these reference standards measure. Without harmonized methodological frameworks, cross-study comparisons risk conflating true model capability with study-specific design choices.

### 4.3 Gaps and suggestions on clinical implementation pathways

Despite strong technical performance, translation into routine clinical practice remains limited. Most studies prioritized algorithmic accuracy over prospective validation, workflow integration, or regulatory considerations. End-to-end deep learning models dominate the field due to their efficiency and automation, yet their limited interpretability may hinder clinician trust and adoption, particularly in screening contexts where false positives can have downstream consequences.

To bridge this gap, future research should build on our findings to address three critical gaps:

First, standardization: Adopt consensus protocols for ROI selection (e.g., L1-L4 for diagnosis, T7-L1 for chest CT screening) and dual reference standards (DXA + QCT) to enable cross-study benchmarking. Publicly available annotated datasets with standardized imaging parameters would accelerate this alignment.

Second, context-aware model design: Match workflows to clinical purpose—HU-based/end-to-end models for screening, radiomics for early diagnosis, and multimodal models (integrating imaging, clinical data, and fall risk factors) for fracture prediction—rather than pursuing a “one-size-fits-all” approach. Explainable AI (XAI) should be integrated into end-to-end models to highlight key decision-making features (e.g., HU thresholds, trabecular texture), enhancing clinical acceptance.

Third, prospective multicenter validation: Conduct large-scale studies (n > 10,000) to evaluate real-world performance, particularly for opportunistic screening in lung cancer or abdominal CT programs. Integration with PACS/EMR systems and attention to regulatory compliance are essential for routine deployment.

Ultimately, the greatest value of AI-enhanced CT may lie not in replacing existing standards, but in complementing them—enabling large-scale opportunistic screening, refining risk stratification, and identifying individuals who would benefit most from targeted DXA, QCT, or preventive intervention.

### 4.4 Limitations

Our scoping review has several limitations. First, despite the use of a comprehensive search strategy, relevant studies published in non-English languages or indexed in non-major databases may have been missed. Second, considerable heterogeneity across included studies—particularly in CT acquisition parameters, reference standards, and AI modeling approaches—limited direct comparison and precluded quantitative synthesis. Third, this review focused solely on CT-based applications and did not include AI studies using other imaging modalities, such as X-ray or MRI, which may offer complementary insights. Finally, most included studies were retrospective and single-center, potentially limiting the generalizability of our findings to broader populations and real-world clinical settings.

## 5 Conclusion

This scoping review identifies three principal application areas of CT-based artificial intelligence in osteoporosis—diagnosis, opportunistic screening, and fracture-risk prediction. AI-enhanced CT already demonstrates diagnostic and screening performance comparable to DXA and QCT. Fracture-risk prediction, although clinically more meaningful, requires further optimization through multimodal data integration. Nonetheless, heterogeneous methodologies and the absence of standardized processing pipelines or clinical validation frameworks continue to limit comparability and translation. Integrating AI directly into routine CT workflows has the potential to substantially reduce the cost of osteoporosis detection, promote earlier identification and treatment, and ultimately lessen the global burden of osteoporotic disease.
